# True Left Bundle Branch Block by Strauss Criteria: Impact on CRT Response and Conduction System Pacing Trial Design—A Systematic Evidence Synthesis Toward Personalized Patient Selection

**DOI:** 10.3390/jpm16070389

**Published:** 2026-07-21

**Authors:** Athanasios Saplaouras, Panagiotis Mililis, Stavroula Koskina, Athanasios Makris, Sokratis Oikonomou, Vasileios Cheilas, Theodoros Efremidis, Athena Batsouli, Ourania Kariki, George Bazoukis, Sotirios Xydonas, Theodoros Karamitsos, Christodoulos Papadopoulos, Nikolaos Fragakis, Michael Efremidis, Konstantinos P. Letsas

**Affiliations:** 1 Department of Cardiology, Onassis Hospital, 17674 Athens, Greece; p.mililis@onasseio.gr (P.M.); s.koskina@onasseio.gr (S.K.); a.makris@onasseio.gr (A.M.); s.oikonomou@onasseio.gr (S.O.); v.cheilas@onasseio.gr (V.C.); t.efraimidis@onasseio.gr (T.E.); o.kariki@onasseio.gr (O.K.); m.efraimidis@onasseio.gr (M.E.); 2 Second Department of Cardiology, Hippokration General Hospital, Aristotle University of Thessaloniki, 54124 Thessaloniki, Greece; tkaramitsos@auth.gr (T.K.); chpapado@auth.gr (C.P.); nfrag@auth.gr (N.F.); 3 Department of Cardiology, Evaggelismos Hospital, 10676 Athens, Greece; athmpatsouli@gmail.com (A.B.); sotxyd@gmail.com (S.X.); 4 Department of Cardiology, General Hospital Larnakas, Larnaka 6301, Cyprus; gbazoykis@yahoo.gr

**Keywords:** cardiac resynchronization therapy, left bundle branch block, Strauss criteria, CRT response, conduction system pacing, left bundle branch pacing

## Abstract

**Background/Objectives**: About one-third of patients who receive cardiac resynchronization therapy (CRT) do not show meaningful clinical improvement. One possible reason is that current criteria for diagnosing left bundle branch block (LBBB) do not clearly separate true conduction block from other conditions that can produce a similar QRS pattern, since they rely mainly on surface ECG features. The Strauss criteria have been proposed as a stricter alternative. They include sex-specific QRS duration thresholds and require a mid-QRS notch or slur, with the goal of better identifying patients whose conduction abnormality may be correctable. Selecting candidates on this basis reflects a personalized-medicine approach that matches resynchronization to the individual conduction substrate rather than applying a single wide-QRS threshold to all patients. In this review, we assess whether LBBB defined by the Strauss criteria is linked to better CRT outcomes, and we examine how these criteria have been used in the design of randomized clinical trials investigating conduction system pacing (CSP). **Methods**: A systematic search of PubMed, EMBASE, and the Cochrane Library was conducted (last updated April 2026) in accordance with PRISMA 2020 guidelines. Evidence was drawn from two groups of studies: (1) observational studies comparing CRT outcomes in Strauss-positive versus Strauss-negative patients, and (2) randomized CSP trials conducted in populations enriched using either Strauss-type or typical LBBB morphology. **Results**: A total of 17 comparative studies (*n* ≈ 4200) were included in Part 1; most (14 of 17) reported outcomes favouring Strauss-defined LBBB, with the greatest signal in non-ischemic cardiomyopathy, although these were mostly small retrospective studies with heterogeneous outcome definitions. However, two large registry analyses found no incremental benefit of strict over conventional criteria. In Part 2, six randomized controlled trials were analyzed. The HeartSync-LBBP trial (*n* = 200, 36-month follow-up) demonstrated significantly lower rates of mortality or heart failure hospitalization with LBBP compared to BiVP (8% vs. 28%; HR 0.26; 95% CI 0.12–0.57), with 98% technical success for LBBP. In contrast, the PhysioSync-HF trial (*n* = 173) showed superiority of BiVP, with CSP success of only 69% and 42.8% of procedures performed by less experienced operators. Similarly, the LEFT-BUNDLE-CRT trial (*n* = 176) found that LBBAP did not meet non-inferiority criteria compared to BiVP (RR 0.95; 95% CI 0.88–1.02). **Conclusions**: Strauss-defined LBBB is associated with improved CRT outcomes in observational studies, particularly in non-ischemic cardiomyopathy; this reflects an association rather than established superiority or causal benefit, and no randomized trial has yet compared Strauss-guided against guideline-based patient selection. In randomized CSP trials, both operator experience and patient selection appear to be critical determinants of success. BiVP remains effective even in patients meeting strict Strauss criteria. Further research is needed to determine whether the application of Strauss criteria improves outcomes beyond current guideline-based patient selection.

## 1. Introduction

Two decades ago, landmark trials showed that cardiac resynchronization therapy (CRT) with biventricular pacing (BiVP) reduces the risk of death and heart failure–related hospitalization in patients with a wide QRS complex [1,2,3,4,5]. However, a considerable proportion of patients still do not respond to CRT, with about one-quarter to one-third experiencing no meaningful clinical benefit. This rate has changed little since those early trials, despite ongoing improvements in lead design, device technology, and programming strategies [6,7,8].

An important and often overlooked issue is that the criteria currently used to diagnose left bundle branch block (LBBB) were not designed to distinguish true conduction system failure from other conditions that can produce similar QRS patterns. For example, QRS widening due to diffuse myocardial fibrosis, partial conduction delay, or left ventricular hypertrophy with strain may all meet guideline-defined LBBB criteria, yet may not result in the electrical dyssynchrony that CRT is intended to correct [9,10,11]. These patients may undergo an invasive procedure from which they are unlikely to benefit, and their inclusion in clinical trials can dilute the apparent treatment effect in patients with true conduction block. Refining who is offered CRT is therefore a question of personalized medicine—individualising device therapy to a patient’s specific conduction substrate rather than applying uniform wide-QRS criteria—an approach increasingly emphasised for resynchronization and conduction system pacing and reflected in the most recent physiologic-pacing guidance [12,13].

In 2011, Strauss et al. proposed an alternative approach to defining true complete LBBB. Instead of relying on ECG features associated with CRT response, they based their definition on the expected electrical pattern of complete interruption of the left bundle branch. Their criteria included a QRS duration ≥ 140 ms in men or ≥130 ms in women, the presence of mid-QRS notching or slurring in at least two contiguous leads, and a QS or rS pattern in lead V1. Using these criteria, it was estimated that roughly one in three patients who met conventional ECG definitions of LBBB did not have true complete block and were therefore less likely to benefit from CRT [14]. How LBBB is defined therefore materially changes which patients are identified as CRT candidates, a point underscored by recent analyses of the left bundle branch block criteria in the 2021 European guidelines [15].

This review explores the clinical evidence that has emerged since, focusing on two related but distinct questions. First, whether LBBB defined by the Strauss criteria is associated with improved CRT outcomes compared with conventional definitions—an issue examined in 17 comparative studies summarized in Table 1. Second, how these criteria have been incorporated into the design of randomized trials of conduction system pacing (CSP), and what insights these trials provide—addressed through six completed randomized controlled trials (RCTs) summarized in Table 2.

These two lines of evidence are complementary. Observational studies indicate that Strauss-defined LBBB may better identify patients who are more likely to benefit from resynchronization, and more recent CSP trials have increasingly enriched their populations with patients exhibiting Strauss-type, typical or otherwise physiologically correctable LBBB morphology. The study by Saplaouras et al. (ESC Heart Failure, 2025), represents the authors’ own previously published work and is discussed below on the same objective terms, and by the first author’s name, as the other included studies [32].

## 2. Methods

### 2.1. Search Strategy

The review was conducted according to PRISMA 2020 recommendations [39], the systematic review and meta-analysis was not prospectively registered. We performed a systematic search of PubMed, EMBASE, and the Cochrane Library using combinations of the terms “Strauss,” “strict LBBB,” “true LBBB,” “complete LBBB,” “CRT,” “CRT response,” “CSP,” and “LBBP,” without language restrictions. We also screened the reference lists of all relevant articles. The initial search was carried out in December 2025 and updated in April 2026.

This review is reported in accordance with PRISMA 2020 but is best characterised as a systematic evidence synthesis rather than a conventional systematic review with quantitative meta-analysis: it was not prospectively registered, no formal grading of the certainty of evidence (e.g., GRADE) was undertaken, and no meta-analysis was performed because the marked heterogeneity in study designs, populations, LBBB definitions and outcome measures precluded valid statistical pooling. The complete Boolean combination applied to each database, adapted to its syntax, was: (“Strauss” OR “strict LBBB” OR “true LBBB” OR “complete LBBB”) AND (“cardiac resynchronization therapy” OR “CRT” OR “CRT response” OR “conduction system pacing” OR “CSP” OR “left bundle branch pacing” OR “LBBP”).

With respect to study quality, 16 of the 17 observational studies scored 7–9 on the Newcastle–Ottawa Scale (high quality; range 6–9, median 8), with only one cohort rated moderate (6/9); the most common limitations were retrospective, single-center design and, in the smaller cohorts, limited adjustment for confounders. Among the six randomized trials, five were rated “some concerns” on the Cochrane RoB 2 tool—chiefly reflecting the structurally unavoidable lack of operator blinding in device-implantation trials (domain D2)—and one (HeartSync-LBBP) was at low risk of bias, with none at high risk. Detailed ratings are provided in Table S1 (Newcastle–Ottawa Scale) and Table S2 (Cochrane RoB 2). The full line-by-line search strategy for each database (Table S3) and the completed PRISMA 2020 checklist (Table S4) are provided as Supplementary Material.

Titles and abstracts were screened independently by two reviewers (A.S. and P.M.), and all potentially relevant studies were reviewed in full. Any disagreements were resolved through discussion with a third reviewer (K.P.L.). After removing duplicates, 312 records remained; 270 were excluded during title and abstract screening, leaving 42 studies for full-text review. Of these, 18 were excluded: 7 due to ineligible study design, 6 because they did not report outcomes that could be compared using Strauss criteria, 3 due to overlapping cohorts, and 2 because they applied Perrin criteria without including Strauss criteria.

**Figure 1:**
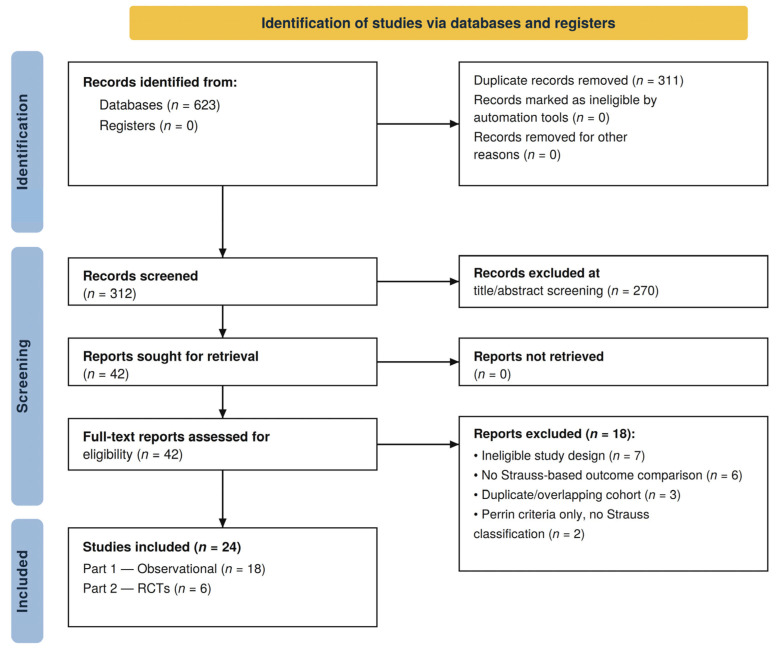
PRISMA 2020 flow diagram showing the identification, screening, eligibility assessment, and inclusion of studies in this systematic evidence synthesis.

In total, 24 studies were included. Part 1 consisted of 18 studies (17 comparative studies plus the original 2011 criteria paper), while Part 2 included 6 randomized controlled trials, with LEFT-BUNDLE-CRT added as the sixth RCT following the April 2026 update. Because of differences in study design, patient populations, and endpoints, a formal meta-analysis was not feasible. Instead, the findings are presented as a structured narrative synthesis (Figure 1).

#### 2.1.1. Part 1: Studies Evaluating Strauss-Defined LBBB and CRT Outcomes (Table 1)

Studies were included if they met three main criteria: use of strict morphological ECG criteria to define left bundle branch block (LBBB), enrollment of patients undergoing CRT or CRT-D, and reporting at least one relevant outcome comparing Strauss-positive and Strauss-negative groups (such as echocardiographic findings, heart failure hospitalization, ventricular arrhythmias, or all-cause mortality). Case reports and editorials were excluded. Study quality was assessed using the Newcastle–Ottawa Scale (see Table S1) [40].

#### 2.1.2. Part 2: Randomized Trials of CSP in Populations Enriched for Strauss-Type or Typical LBBB Morphology (Table 2)

Randomized controlled trials were included if they enrolled patients with Strauss-defined or Strauss-type/typical left bundle branch block morphology, compared conduction system pacing (His bundle pacing or left bundle branch area pacing) with BiVP or another control strategy, and reported at least one pre-specified cardiac outcome. Studies based only on QRS duration, without selection for the relevant morphology, were excluded.

PhysioSync-HF was included as a predefined exception. Although enrollment was based on QRS duration and did not require Strauss criteria, most patients in both arms (96.6% in the CSP group and 94.2% in the BiVP group) had Strauss-type or typical LBBB morphology at baseline [37]. This was determined retrospectively and should be considered a limitation; its results are best interpreted alongside rather than equivalent to studies that used Strauss criteria as a formal enrollment standard. Ongoing studies meeting these criteria are reported separately. Study quality was assessed using the Cochrane risk-of-bias tool (Table S2) [41].

Terminology note. Several related terms are used throughout the literature, and it is important to keep their differences in mind. “Strauss-defined LBBB” refers specifically to cases classified using the original Strauss ECG criteria, which include sex-specific QRS duration thresholds and the presence of mid-QRS notching or slurring.

In contrast, “Strauss-type” or “typical” LBBB morphology is used more loosely to describe ECG patterns that resemble those described by Strauss, even if the formal criteria were not applied. In many studies, these cases were identified retrospectively or drawn from populations that were not prospectively selected using Strauss criteria, which can affect consistency.

The term “physiologically correctable” LBBB refers to cases in which the conduction abnormality can be corrected with His-bundle pacing or left bundle branch area pacing. These are related but distinct concepts and should not be used interchangeably in this review.

## 3. Results

### 3.1. Part 1: Strauss Criteria as Predictors of CRT Response—Overview (2011–2025)

Seventeen studies compared CRT outcomes between Strauss-positive and Strauss-negative patients (Table 1), in addition to the original 2011 criteria paper. Altogether, these studies included roughly 4200 patients. Six were prospective cohorts [17,18,19,21,29,30], while the remaining eleven were retrospective or registry-based analyses. Study sizes varied widely (from 37 to 1492 patients), as did follow-up, which ranged from 6 months to 9 years.

Most studies pointed in the same direction. Fourteen of the 17 reported better outcomes in Strauss-positive patients. Bertaglia et al. did not find any clear advantage of strict over conventional LBBB criteria [22], and both van Stipdonk et al. and Shoman et al. reported results that varied depending on the endpoint analyzed [28,30]. It is worth noting that none of these studies was designed to test Strauss-based selection prospectively against guideline-based criteria, so their findings do not directly answer how this approach would perform in routine clinical practice.

#### 3.1.1. Electrophysiological Validation: The Risum Study

Before drawing conclusions from these data, a more basic question needs to be considered: do the Strauss ECG criteria actually identify true conduction block? This was explored by Risum et al. in a 2013 prospective study of 66 patients [17]. They compared strict ECG classification with echocardiographic identification of LBBB as an independent reference. Among the 45 patients who met Strauss criteria, 82% responded at six months, compared with 21% of those who did not. Sensitivity was 90% and specificity was 65%.

The most clinically relevant finding came from the concordance analysis. When both ECG and echocardiography indicated that LBBB was absent, 94% of patients failed to respond to CRT. This high negative predictive value is clinically meaningful, as it suggests that patients meeting neither criterion are unlikely to benefit and may not be good candidates for CRT.

#### 3.1.2. Early Observational Evidence (2012–2015)

Mascioli et al. were among the first to apply the Strauss criteria in a clinical CRT cohort. In 111 patients, they found that true LBBB, defined by Strauss morphology, was independently associated with better echocardiographic response, clinical improvement, and survival [16]. Around the same time, Tian et al. reported similar findings in a smaller cohort of 58 patients: true complete LBBB by Strauss-type criteria was an independent predictor of super-response (OR 11.68; 95% CI 1.97–69.4; *p* = 0.007), and all patients meeting the strict criteria responded to CRT [18]. Although these studies were relatively small, the consistency between them was notable and helped drive further interest in the criteria.

Emerek et al., in a study of 49 patients, showed that strict LBBB criteria more accurately identified significant left ventricular activation delay than conventional definitions, with the difference greater in ischemic (responder proportion 62% to 78%) than in dilated cardiomyopathy (90% to 95%) [20]. Jackson et al. approached the problem differently, using cine CMR to classify contraction patterns within a strict LBBB population. All 19 patients with a type II (U-shaped septal) contraction pattern showed reverse remodeling, compared with 6 of 18 (33%) with a type I pattern (*p* < 0.01); super-response rates were 84% versus 28% [19]. Taken together, these findings suggest that ECG classification and imaging are likely capturing the same underlying substrate from different perspectives. This was explored more formally by Risum et al. in 2015. In a cohort of 208 patients followed for four years, adding echocardiographic contraction pattern assessment to strict ECG classification improved the C-statistic for predicting death, LVAD implantation, or transplantation from 0.63 to 0.71 (HR 3.13 for absence of a contraction pattern; 95% CI 1.64–5.88; *p* < 0.005) [21].

#### 3.1.3. Larger Cohorts and Comparative Definition Studies (2017–2020)

Bertaglia et al. analyzed 335 patients from the CRT MORE registry and did not find a clear advantage of Strauss-like morphology over conventional LBBB criteria (response 65% vs. 59%, *p* = 0.267) [22]. As a registry study, it has the usual limitations, but given its size, the findings are difficult to ignore. In contrast, Caputo et al. examined five LBBB definitions in 316 patients and found that Strauss criteria were independently associated with the composite outcome of death or heart failure hospitalization (HR 0.57; 95% CI 0.40–0.80) [25].

Jastrzębski et al. compared four definitions—ESC, Marriott, WHO/AHA, and Strauss—in 552 CRT patients followed for nine years. The proportion of patients classified as LBBB varied widely depending on the definition used (63.4% for conventional vs. 40.9% for Strauss). During follow-up, 232 patients died and 292 reached the combined endpoint of death or heart failure admission. Among the four definitions, Strauss criteria showed the strongest association with outcomes, with the lowest hazard ratio for all-cause mortality (HR 0.51) [26]. García-Seara et al., studying 198 patients, reported greater reductions in LVESV (−27.6% vs. −19.7%, *p* = 0.04) and larger improvements in LVEF (10.8 vs. 5.1 percentage points, *p* = 0.03) in patients meeting strict LBBB criteria. There was no meaningful difference between Strauss and Perrin definitions [23,42]. Hadjis et al. found similar patterns in 231 patients: QRS duration shortened after CRT in those with strict LBBB (−20.9 ± 12.4 ms) but not in those with conventional LBBB (+6.7 ± 19.4 ms, *p* < 0.0001), and strict LBBB was independently associated with lower mortality (OR 0.49; 95% CI 0.24–0.99; *p* = 0.046) [27].

Kashtanova et al., in a smaller cohort of 39 patients, showed that strict LBBB was associated with better-preserved baseline global longitudinal strain and more pronounced mechanical dyssynchrony before implantation, which in turn was linked to better echocardiographic response at six months [24]. Although the sample size was limited, this fits with earlier observations that strict ECG criteria reflect the degree of underlying dyssynchrony.

Van Stipdonk et al. analyzed the largest dataset in this field (1492 patients) and found that no single LBBB definition was clearly superior across all endpoints [28]. This study is often cited as evidence against the Strauss criteria, but the results are more nuanced. All four definitions were significantly associated with the primary endpoint (LVAD, transplantation, or all-cause mortality), with similar relative risk reductions (39–43%). For Strauss-defined LBBB, the hazard ratio was 0.61 (95% CI 0.51–0.73). Only 13.8% of patients met all four definitions at the same time, highlighting how much the classifications differ. Notably, the ECG features most strongly linked to outcomes—QS or rS in V1, notching or slurring in lateral leads, and absence of Q waves in lateral leads—closely match the components of the Strauss criteria.

#### 3.1.4. Arrhythmia Prediction (2021, 2025)

Most earlier studies focused on mechanical outcomes. Bouazzi et al. looked beyond this, asking whether Strauss criteria might also relate to ventricular arrhythmic risk after CRT. In a prospective cohort of 206 patients followed for two years, absence of a typical LBBB contraction pattern was independently associated with appropriate ICD therapy (HR 1.89; 95% CI 1.04–3.44; *p* = 0.036), whereas strict ECG criteria alone were not [29]. Patients who lacked both Strauss ECG features and the typical contraction pattern had the highest risk, with a 3.3-fold increase in ventricular arrhythmias (HR 3.34; 95% CI 1.75–6.94; *p* < 0.001).

A similar signal was seen in the study by Saplaouras et al. Lower ventricular arrhythmia burden in Strauss-defined LBBB was observed in patients with non-ischemic cardiomyopathy (*p* = 0.049), with no significant difference in those with ischemic cardiomyopathy, suggesting that the effect depends on the underlying substrate [32]. Overall, strict LBBB classification appears to carry information beyond predicting CRT response, with potential implications for ICD management after implantation.

#### 3.1.5. Contemporary Evidence (2022–2025)

In a prospective cohort, Shoman et al. studied 67 patients and found that those with Strauss-defined LBBB had greater improvements in NYHA class, LVEF, LVESV, and global strain at six months. However, there was no significant difference in binary echocardiographic response (*p* = 0.463) [30]. The small sample size—particularly the limited number of Strauss-positive patients—likely reduced the ability to detect a difference in response rates.

Mugnai et al. analyzed 236 patients to examine whether the individual components of the Strauss criteria add predictive value beyond QRS duration alone [31]. In multivariable analysis, only LVEF (OR 0.92) and the QRS-duration threshold (OR 3.70) remained independent predictors; overall, Strauss criteria were associated with response (sensitivity 71.3%, specificity 64.1%). Adding the absence of an S wave in leads V5–V6 improved specificity to 82.6% and positive predictive value to 90.5%. In contrast, a higher Selvester score was associated with a lower likelihood of response (OR 0.818; 95% CI 0.75–0.89; *p* < 0.001), highlighting the impact of myocardial scar.

The study by Saplaouras et al. included 82 patients from two Greek centers, of whom 46 (56.1%) met Strauss criteria [32]. Over a mean follow-up of 52 months, Strauss-defined LBBB was associated with higher CRT response rates (78.3% vs. 27.8%; *p* < 0.01), fewer heart failure hospitalizations (*p* < 0.0001), and lower mortality (17.4% vs. 36.1%; *p* = 0.0475). A lower ventricular arrhythmia burden was also observed, but only in patients with non-ischemic cardiomyopathy (*p* = 0.049).

These benefits were largely seen in the non-ischemic group, where 30 of 33 St-LBBB patients with non-ischemic cardiomyopathy (91%) responded, compared with 6 of 13 (46%) among those with ischemic cardiomyopathy. In the ischemic subgroup, Strauss status was not clearly linked to differences in arrhythmic burden or mortality. This supports the idea that Strauss-defined LBBB reflects a distinct substrate, with the greatest benefit seen when myocardial contractile reserve is preserved. Individualising selection by substrate in this way—combining refined ECG criteria with quantitative, observer-independent measures such as vectorcardiographic QRS area—is precisely the kind of precision-medicine strategy that could translate the observed association into actionable patient selection [43].

One additional study provides some supporting context, although it did not meet the inclusion criteria for this analysis. In a single-arm pilot study of 54 patients, Atabekov et al. included only patients with Strauss-defined LBBB and did not include a comparison group [44]. In this cohort, 72.2% were classified as super-responders. A combined model using S-wave amplitude in V2, global longitudinal strain, and interventricular delay showed strong predictive performance (AUC 0.957; sensitivity 87.2%; specificity 100%). The study was not included in Table 1 because it lacked a Strauss-negative comparator group.

### 3.2. Part 2: Randomized Trials of CSP in Populations Enriched for Strauss-Type or Typical LBBB Morphology—Overview

The use of Strauss criteria in CSP trials reflects a simple idea: if resynchronization works best when pacing at or beyond a proximal conduction block, then it makes sense to study patients who actually have that type of block, rather than those with nonspecific QRS widening. Six randomized trials have now reported results in populations enriched for Strauss-type or typical LBBB morphology (Table 2).

#### 3.2.1. His-Alternative

The His-Alternative trial was the first randomized study to use Strauss-defined LBBB as its only enrollment criterion. It included 50 patients with LVEF ≤ 35%, randomized 1:1 to His bundle–corrective CRT or conventional BiVP. In the His-CRT group, conduction correction was achieved in 72% of patients. There was no significant difference in the intention-to-treat analysis. However, among patients in whom corrective capture was achieved, outcomes were better, with higher LVEF (48% vs. 42%, *p* < 0.05) and lower LVESV (65 vs. 83 mL, *p* < 0.05) compared with BiVP [33]. The study was small, and the fact that nearly one-third of patients did not achieve corrective capture likely diluted the overall result. Still, the per-protocol findings suggest that pacing closer to the site of block may be more effective than pacing around it, even if the study was not powered to prove this.

#### 3.2.2. LBBP-RESYNC

LBBP-RESYNC was the first randomized trial to compare left bundle branch pacing with BiVP in a population selected using Strauss criteria [34]. Forty patients with non-ischemic cardiomyopathy, Strauss-defined LBBB, and LVEF ≤ 40% were randomized 1:1 to LBBP or BiVP and followed for six months. LBBP led to a greater increase in LVEF (mean difference 5.6%; 95% CI 0.3–10.9; *p* = 0.039), along with larger reductions in LVESV and lower NT-proBNP levels. Technical success was 90%. Other outcomes—NYHA class, 6 min walk distance, QRS duration, and overall response—were similar between groups. Restricting enrollment to non-ischemic patients with Strauss-defined LBBB helped create a more uniform study group, although it also limits how broadly the results can be applied.

#### 3.2.3. LEVEL-AT

LEVEL-AT enrolled 70 patients with a standard indication for CRT, including a subgroup with Strauss-defined LBBB, and randomized them to CSP (His bundle pacing or LBB area pacing) or BiVP [35]. The primary endpoint was change in left ventricular activation time at 45 days, assessed using electrocardiographic imaging, with a non-inferiority design. About 23% of patients assigned to CSP crossed over to BiVP due to unsuccessful lead placement. Despite this, CSP met the non-inferiority criterion for the primary endpoint and produced similar reverse remodeling and clinical outcomes at six months. This suggests that when it can be performed successfully, conduction system pacing achieves resynchronization comparable to BiVP, although feasibility remains a limitation.

#### 3.2.4. HeartSync-LBBP

HeartSync-LBBP enrolled 200 patients with LBBB and LVEF ≤ 35% across six centers in China, randomizing them to LBBP or BiVP, with a median follow-up of 36 months [36]. One of the main strengths of the study was the procedural approach: LBBB correction was confirmed during implantation rather than assumed from the ECG. Success rates were high in both groups (98% for LBBP and 94% for BiVP), and all operators had extensive experience with CSP. Most patients (82.5%) had non-ischemic cardiomyopathy.

The primary endpoint—death or heart failure hospitalization—occurred less often in the LBBP group (8% vs. 28%; HR 0.26; 95% CI 0.12–0.57), mainly due to fewer hospitalizations for heart failure (7% vs. 28%). Mortality alone was similar between groups. These findings suggest that, when the procedure is performed successfully and in experienced hands, LBBP can lead to better long-term outcomes.

#### 3.2.5. PhysioSync-HF

PhysioSync-HF enrolled 173 patients across 14 centers in Brazil and randomized them to CSP or BiVP, with 12 months of follow-up. Patients had LBBB with QRS ≥ 130 ms and LVEF ≤ 35%. Strauss criteria were not required, although most patients in both groups had a typical Strauss-type LBBB pattern [96.6% (CSP) and 94.2% (BiVP)] at baseline [37]. The primary outcome—a composite of death, heart failure hospitalization, urgent HF visits, and change in LVEF—favored BiVP (OR 2.36; 95% CI 1.37–4.06). CSP did not meet non-inferiority and instead showed a significant difference between groups (*p* = 0.002). A time-to-event analysis showed a similar trend, with outcomes numerically worse in the CSP group (HR 2.35; 95% CI 0.99–5.61). LVEF improved in both groups but remained higher with BiVP at 12 months (39% vs. 35%; mean difference 3.8%; 95% CI 0.3–7.3%). Other clinical measures improved to a similar extent in both groups.

The procedural setting was quite different from HeartSync-LBBP. CSP success was achieved in 69% of patients, and some patients received deep septal pacing instead of confirmed left bundle capture. In addition, many procedures were performed by operators with limited experience. These factors likely influenced the results.

#### 3.2.6. LEFT-BUNDLE-CRT

LEFT-BUNDLE-CRT enrolled 176 patients across several centers in Spain, all with a class I or IIa indication for CRT and Strauss-defined LBBB [38]. Patients were randomized to LBBAP (*n* = 92) or BiVP (*n* = 84). The primary endpoint was CRT response at six months, defined as either improvement in the clinical composite score or a ≥15% reduction in LVESV. Crossovers occurred in 26 patients (14.9%).

Response rates were high in both groups (94.6% with BiVP and 89.7% with LBBAP; RR 0.95; 95% CI 0.88–1.02), and the study did not meet its non-inferiority criterion. The individual components showed a similar pattern, with slightly greater improvement in the BiVP group, while adverse events and heart failure hospitalizations were similar between groups.

Overall, these findings are consistent with those from PhysioSync-HF. In patients with Strauss-defined LBBB, LBBAP was not shown to be non-inferior to BiVP when using a response-based endpoint, although both approaches were associated with high response rates and broadly similar clinical outcomes.

## 4. Discussion

### 4.1. Observational Evidence

The available comparative studies show a broadly consistent pattern. Patients meeting Strauss criteria have a more complete form of left bundle conduction failure, more severe pre-implant mechanical dyssynchrony, and a substantially higher probability of CRT response. The pattern holds across independent groups from Greece, Denmark, Poland, Russia, Canada, Spain, Egypt, Italy, and Switzerland, across follow-up periods ranging from six months to nine years, and across endpoints ranging from echocardiographic remodeling to all-cause mortality and ventricular arrhythmias. Most studies showed a consistent direction of effect, although publication bias and differences in endpoints remain important limitations.

However, these studies do not establish whether selecting patients prospectively using Strauss criteria improves outcomes compared with current guideline-based selection—that question has not been tested in a randomized trial. Bertaglia and van Stipdonk, whose datasets are the largest in the literature, both failed to demonstrate that strict criteria outperform conventional ones in practice [22,28]. Registry analyses have limitations; they offer generalizability that single-center cohorts cannot match. The association between Strauss-defined LBBB and improved outcomes is consistent across studies. Whether that association translates into a prospective selection tool that outperforms guideline-based selection is a separate, as yet untested, claim.

### 4.2. Non-Ischemic Cardiomyopathy: Where Strict Criteria Matter Most

The concentration of benefit in non-ischemic patients is mechanistically predictable. In ischemic cardiomyopathy, scar limits the contractile response to resynchronization regardless of how precisely the conduction abnormality has been characterized; correcting the electrical sequence does not help tissue that cannot contract. In non-ischemic disease, where the conduction abnormality is often the dominant driver of dyssynchrony and the myocardium retains contractile reserve, accurate substrate identification makes a proportionally larger difference to outcome. The clinical case for Strauss assessment is strongest in this subgroup. In non-ischemic cardiomyopathy, Strauss-defined LBBB seems to identify patients in whom dyssynchrony is mainly conduction-driven and myocardial contractile reserve is preserved. This mechanistic distinction has implications for trial design: a selection strategy trial would have greatest power—and most clinical relevance—if stratified by aetiology.

### 4.3. Randomized Trial Evidence

The six trials addressed related but not identical clinical questions, and their findings are easier to interpret when considered in sequence. His-Alternative established that the substrate identified by Strauss criteria can be corrected by conduction system pacing in a substantial proportion of patients, though the 72% technical success rate foreshadowed the execution problems that would surface in later trials. LBBP-RESYNC demonstrated that in a deliberately homogeneous non-ischemic Strauss population, correcting the block with LBBP produced better echocardiographic outcomes than BiVP at six months [34]. LEVEL-AT confirmed that electrical resynchronization with CSP is comparable to BiVP, while its 23% crossover rate was a candid acknowledgement that technical success cannot be taken for granted [35]. HeartSync-LBBP, with 200 patients, 36 months of follow-up, 98% technical success, and LBBB correction confirmed at implantation, provides the strongest clinical evidence to date, with 8% versus 28% for the primary endpoint [36]. PhysioSync-HF enrolled a broadly similar LBBB population (96.6% CSP-arm and 94.2% BiVP-arm patients had Strauss-type LBBB at baseline) [37]; however, CSP was technically successful in only 69% of cases, and 42.8% of implants were performed by operators with fewer than 40 prior CSP cases. In this context, BiVP was associated with superior outcomes. LEFT-BUNDLE-CRT, a multi-center Spanish trial in 176 Strauss-defined LBBB patients randomized to LBBAP or BiVP, reported in April 2026 that LBBAP did not meet non-inferiority for CRT response at six months (89.7% vs. 94.6%; RR 0.95; 95% CI 0.88–1.02), though both arms achieved high response rates and comparable clinical outcomes including heart failure hospitalization [38].

PhysioSync-HF does not appear to be primarily limited by patient selection; rather, outcomes were likely influenced by variability in CSP delivery, procedural success, and operator experience. Identifying the right substrate and executing the correction are two distinct requirements; the trial met the first without reliably meeting the second. Left bundle branch area pacing follows a steep learning curve, and the outcome data of PhysioSync-HF reflect that curve directly. Differences in procedural success and operator experience likely account for some of the variation across trials, but the findings of LEFT-BUNDLE-CRT suggest that these factors do not fully explain the results. In a strictly Strauss-defined population with adequate enrollment rigor, LBBAP still failed to demonstrate non-inferiority to BiVP. This suggests that even optimal substrate selection does not guarantee CSP superiority, and that BiVP is a highly effective and consistently deliverable strategy even in physiologically ideal substrates. The hypothesis that CSP is intrinsically superior for true LBBB remains unconfirmed by randomized evidence. Larger real-world datasets and recent meta-analyses of left bundle branch area pacing versus biventricular pacing point in a similar direction, reporting comparable or better resynchronization with conduction system pacing while leaving the question of Strauss-based selection unanswered [45,46]. Importantly, none of the available randomized trials were designed to test Strauss-based selection against guideline-based selection; rather, they evaluated pacing modality within populations already enriched for physiologically correctable LBBB.

### 4.4. Reproducibility and the Path to Implementation

The practical barrier to wider adoption of Strauss criteria is not conceptual but operational. Mid-QRS notching and slurring require deliberate visual attention and generate genuine disagreement between experienced readers, particularly when QRS duration or notch depth is borderline. Van Stipdonk et al. documented substantial inter-observer variability when different raters applied different criteria to identical ECGs [47]. Findings observed in expert settings may not translate directly into routine clinical practice. In the short term, involving an electrophysiologist in reviewing borderline ECGs before the implant decision is made provides a pragmatic safeguard. Longer-term, deep learning classifiers trained and validated on well-annotated Strauss datasets could make morphological classification as reproducible as automated QRS duration measurement [48].

Beyond refined morphology, a complementary strategy is to replace dichotomous visual classification with an objective, continuous measure of ventricular activation delay. The reader-dependence of morphological classification is greatest for precisely the features that define Strauss LBBB, namely mid-QRS notching or slurring and the sex-specific duration thresholds, and it extends to procedural assessment during left bundle branch area pacing [49]. Vectorcardiographic (VCG) QRS area, derived automatically from the 12-lead ECG-synthesised vectorcardiogram, is not subject to the intra- and inter-observer variability that affects these judgements, and it can be analyzed as a continuous variable rather than a black-or-white category. In its original description QRS area predicted echocardiographic response substantially better than QRS duration or morphology (odds ratio approximately 10.2 versus 2.5 and 5.5, respectively) [50], and in a large multicenter cohort it was an independent determinant of the composite clinical outcome after CRT [51]. QRS area therefore represents a reproducible adjunct to Strauss classification and shares the same objective, observer-independent rationale as the deep-learning approaches noted above [48].

The electrical substrate captured by Strauss criteria is only one of several determinants of CRT response and should not be interpreted in isolation. In addition to heart-failure aetiology, discussed above as the dominant modifier, female sex is associated with higher response rates and greater reverse remodelling, in part because women more often have smaller ventricles, true LBBB and non-ischaemic disease and a higher ratio of QRS duration to ventricular size [52,53]. Older age, ischaemic aetiology, larger left ventricular volumes, atrial fibrillation and greater myocardial scar burden attenuate the response. These modifiers, together with the under-representation of women noted in the Limitations, should be weighed alongside ECG morphology when Strauss criteria are applied to individual patients.

Finally, CRT response is better understood as a continuum than as a binary outcome. The widely used threshold of a 15% reduction in left ventricular end-systolic volume oversimplifies a graded phenomenon, and agreement among binary response definitions is itself poor [6]. A more informative benchmark is how closely a patient approaches the maximal reverse remodelling achievable for their substrate. In the most favourable substrate, women with true (Strauss-defined) LBBB and non-ischaemic cardiomyopathy, super-response with left ventricular end-systolic volume reductions on the order of 50% and frequent normalisation of ejection fraction is an appropriate expectation rather than a nominal 15% decrease, and accepting less may signal suboptimal resynchronization. This interpretation is consistent with the high super-response rates reported across the studies reviewed here and further supports both rigorous substrate selection and the use of continuous predictors such as QRS area [18,36,44].

Differences in outcome across the included observational studies may also reflect confounding beyond ECG morphology. Ischaemic versus non-ischaemic aetiology and myocardial scar burden, baseline QRS duration, left ventricular lead position, the adequacy of guideline-directed medical therapy, the use of imaging-guided implantation, and device programming (including atrioventricular and interventricular optimisation and the percentage of effective biventricular pacing) vary substantially between studies and can influence CRT response independently of Strauss classification. Because these variables were inconsistently reported and rarely adjusted for, residual confounding cannot be excluded, and the association between Strauss-defined LBBB and outcome should be interpreted accordingly.

## 5. Limitations

Part 1 includes 17 comparative studies (Strauss-positive versus Strauss-negative patients); one additional single-arm study enrolling only Strauss-positive patients (Atabekov et al., 2025) was identified but excluded from the comparative analyses because it lacks a Strauss-negative comparator group and does not contribute to the positive rate calculation [44]. The vast majority of the 17 comparative studies are retrospective or observational. Publication bias likely inflates the 82% positive rate, and the fact that two of the largest datasets found no incremental benefit is an important counterweight that should not be minimized. The 14-of-17 positive-rate summary is descriptive only: it aggregates studies with markedly heterogeneous endpoints—echocardiographic response, all-cause mortality, HF hospitalization, ventricular arrhythmia burden, and contraction pattern—and should not be interpreted as a pooled estimate of effect consistency. Outcome definitions differ substantially across studies—ranging from six-month echocardiographic response to nine-year mortality—which precludes direct comparison. Women are consistently underrepresented, despite the fact that Strauss criteria incorporate sex-specific QRS thresholds. For Part 2, the six RCTs differ enough in population characteristics, operator experience requirements, CSP modalities, and definitions of technical success that drawing firm cross-trial conclusions carries risk. None of them was designed to test Strauss-based selection against guideline-based selection: they all enrolled populations enriched to varying degrees for Strauss-type, typical, or otherwise physiologically correctable LBBB morphology and then randomized pacing strategy. That is a meaningful limitation when interpreting what the trials say about the value of the selection criteria themselves.

## 6. Conclusions

Observational evidence. Across the 17 comparative studies, Strauss-defined LBBB was generally associated with improved CRT outcomes. Patients meeting Strauss criteria appear to represent a more homogeneous physiological substrate than those classified by broader guideline-based LBBB definitions. One of the most informative studies was the Jastrzębski cohort—nine years of follow-up in 552 patients, with Strauss outperforming three competing definitions on mortality prediction [26]. The mechanistic link was explored by Risum et al., who demonstrated an association between strict ECG classification and the contraction pattern on echocardiography that predicts reverse remodeling [21]. The study by Saplaouras et al. reports the same signal across CRT response rates, heart failure hospitalization, ventricular arrhythmia burden, and mortality [32].

Randomized trial evidence. The six randomized CSP trials add a different but complementary dimension. Morphological enrichment for Strauss-type or typical LBBB has become a standard design feature, built on the premise that true complete conduction block is the substrate most likely to benefit from physiological resynchronization. HeartSync-LBBP demonstrated the potential when substrate identification is rigorous and procedural execution is consistent: LBBP produced a large, sustained reduction in death and HF hospitalization [36]. PhysioSync-HF demonstrated what happens when patient selection is adequate but execution is not: BiVP may have performed better in part because it was more reliably delivered. Both patient selection and operator experience are important [37]. LEFT-BUNDLE-CRT further demonstrates that even with rigorous Strauss-defined substrate selection, LBBAP did not achieve non-inferiority to BiVP, confirming that high BiVP efficacy persists even in physiologically ideal substrates [38].

Clinical implications. Strauss ECG criteria may be useful as an adjunct when LBBB classification is uncertain or when the indication for CRT is borderline, particularly in non-ischemic cardiomyopathy where substrate identification is likely to matter more. For patients being considered for CSP, operator experience with left bundle branch area pacing is also an important factor and should be considered alongside ECG morphology. These are pragmatic suggestions informed by the observational evidence; they have not been tested in prospective selection trials. A key next step would be a prospective randomized trial comparing Strauss-based with guideline-based selection for CRT. Framed in this way, Strauss-based characterization exemplifies the personalized-medicine goal of matching device therapy to the individual conduction substrate, aligning CRT candidate selection with the broader move toward precision cardiovascular care [12].

## Figures and Tables

**Table 1:** Part 1: Studies evaluating Strauss-defined LBBB and CRT outcomes (2011–2025), ordered chronologically.

Study (Year)	Design, *n*, Follow-Up	Key Findings	Verdict
Strauss et al. [14] (2011)	Criteria paper; N/A	Proposed sex-specific thresholds (QRS ≥ 140 ms in men, ≥130 ms in women) combined with mandatory mid-QRS notching or slurring in at least two leads and QS or rS in V1. Estimated that roughly one in three patients labeled as LBBB by conventional criteria did not have true complete conduction block.	Defining
Mascioli et al. [16] (2012)	Observational *n* = 111; long-term (2007–2011)	Patients with true LBBB by Strauss morphology had better echocardiographic response, NYHA improvement, and survival than those who did not. One of the earliest studies to apply strict criteria in a clinical CRT cohort.	Positive
Risum et al. [17] (2013)	Prospective *n* = 66; 6 months	82% response rate in patients meeting Strauss criteria versus 21% in those who did not. When both ECG and echocardiographic LBBB classification agreed that block was absent, non-response occurred in 94% of cases—a negative predictive value high enough to be clinically useful.	Positive
Tian et al. [18] (2013)	Prospective *n* = 58; 6 months	True complete LBBB by Strauss-type criteria was an independent predictor of super-response (OR 11.68; 95% CI 1.97–69.4, *p* = 0.007). Every patient meeting the strict criteria responded to CRT.	Positive
Jackson et al. [19] (2014)	Prospective CMR *n* = 37; 6 months	Among 37 strict LBBB patients, those with a U-shaped (type II) septal contraction pattern on CMR all achieved reverse remodeling (100% vs. 33% in type I, *p* < 0.01). Super-response rates were 84% versus 28% (*p* < 0.01). The finding showed that strict ECG criteria alone do not capture all the response heterogeneity within the strict LBBB population.	Positive
Emerek et al. [20] (2015)	Observational *n* = 49; 6 months	Strict LBBB criteria more accurately identified patients with significant LV activation delay than conventional criteria. The predictive advantage was greater in ischemic disease (responder proportion 62% to 78%) than in dilated cardiomyopathy (90% to 95%).	Positive
Risum et al. [21] (2015)	Prospective 2-center *n* = 208; 4 years	Absence of a typical LBBB contraction pattern on strain echocardiography was independently associated with death, LVAD implantation, or transplantation (HR 3.13; 95% CI 1.64–5.88; *p* < 0.005). Adding echo assessment to strict ECG classification improved the C-statistic from 0.63 to 0.71 (*p* = 0.02), confirming that the two modalities assess the same substrate from different angles.	Positive
Bertaglia et al. [22] (2017)	Multicenter registry *n* = 335; 12 months	In the CRT MORE registry, echocardiographic response rates were 65% in patients with strict LBBB and 59% in those with conventional LBBB (*p* = 0.267). No incremental benefit of applying stricter criteria was demonstrated. The largest neutral dataset in this literature.	Neutral
García-Seara et al. [23] (2018)	Observational *n* = 198; 12 months	Patients meeting Strauss or Perrin strict LBBB criteria had greater LVESV reduction (−27.6% vs. −19.7%, *p* = 0.04) and higher LVEF gain (10.8 vs. 5.1 percentage points, *p* = 0.03) than those who did not. Heart failure hospitalization rates were also lower. There was no difference in outcomes between the two strict definitions.	Positive
Kashtanova et al. [24] (2018)	Observational *n* = 39; 6 months	Patients with strict LBBB had better-preserved baseline global longitudinal strain and more pronounced mechanical dyssynchrony before implantation, which translated into superior echocardiographic response to CRT at six months.	Positive
Caputo et al. [25] (2018)	Multicenter *n* = 316; median 55 months	Among five LBBB definitions tested, Strauss criteria were independently associated with the composite endpoint of death or HF hospitalization in multivariate analysis (HR 0.57; 95% CI 0.40–0.80). The low use of quadripolar LV leads (<5%) limits comparability with current practice.	Positive
Jastrzębski et al. [26] (2018)	Retrospective cohort *n* = 552 (2006–2014); 9 years (232 deaths)	LBBB prevalence varied markedly by definition: conventional 63.4%, Marriott 46.0%, WHO/AHA 39.5%, Strauss 40.9%. Over 9 years, 232 patients died and 292 met the combined endpoint of death or HF admission. The Strauss definition outperformed all three competing definitions, achieving the best C-statistics and the lowest hazard ratio for all-cause mortality (HR 0.51).	Positive
Hadjis et al. [27] (2019)	Observational *n* = 231; 6 months	Patients with strict LBBB showed substantial QRS narrowing after CRT (−20.9 ± 12.4 ms) compared with those with conventional LBBB, who showed no narrowing at all (+6.7 ± 19.4 ms, *p* < 0.0001). Strict LBBB was independently associated with lower all-cause mortality (OR 0.49; 95% CI 0.24–0.99, *p* = 0.046).	Positive
van Stipdonk et al. [28] (2020)	Multicenter retrospective *n* = 1492 (2001–2015); Mean ~3.4 yrs	In the largest single analysis in this literature (1492 patients, follow-up 3.4 ± 2.4 years), 472 (32%) patients experienced the primary endpoint (LVAD, transplantation, or all-cause mortality). All four LBBB definitions were significantly associated with outcome; relative risk reduction ranged from 39% to 43% with no significant difference between definitions. HR for Strauss-defined LBBB: 0.61 (95% CI 0.51–0.73). Only 13.8% of patients met all four definitions simultaneously. The three ECG components most strongly predictive of outcome were QS or rS in V1, notching or slurring in lateral leads, and absent Q waves in lateral leads—the morphological basis of Strauss criteria.	Mixed
Bouazzi et al. [29] (2021)	Prospective 2-center *n* = 206; 2 years	In 206 patients followed for two years after CRT, absence of a typical LBBB contraction pattern was independently associated with ventricular arrhythmia risk (HR 1.89; 95% CI 1.04–3.44, *p* = 0.036). Strict ECG criteria alone were not independently associated with arrhythmic risk. Patients fulfilling neither echocardiographic nor strict ECG criteria for LBBB had a 3.3-fold increase in risk (HR 3.34; 95% CI 1.75–6.94; *p* < 0.001), extending the clinical relevance of strict classification into ICD programming decisions.	Positive
Shoman et al. [30] (2022)	Prospective *n* = 67; 6 months	Strauss-positive patients showed significant improvements in NYHA class, LVEF, LVESV, global longitudinal strain, and global circumferential strain at six months. The overall echocardiographic response rate did not differ significantly between groups (*p* = 0.463), likely due to the limited number of patients in the Strauss group.	Mixed
Mugnai et al. [31] (2022)	Observational *n* = 236; 6–12 months	Strauss criteria were associated with CRT response (sensitivity 71.3%, specificity 64.1%); in multivariable analysis, only LVEF (OR 0.92) and the QRS-duration threshold (OR 3.70) remained independent predictors. Adding absence of S wave in V5–V6 to Strauss criteria improved specificity to 82.6% and positive predictive value to 90.5%. The Selvester	Positive
Saplaouras et al. [32] (2025)	Retrospective 2-center *n* = 82 (ICM 39%, NICM 61%); Mean 52 months (January 2013–June 2021)	St-LBBB patients had significantly higher CRT response rates (78.3% vs. 27.8%, *p* < 0.01), driven primarily by non-ischaemic patients: among St-LBBB, 30/33 (91%) with NICM responded versus 6/13 (46%) with ICM. Fewer HF hospitalizations (*p* < 0.0001) and lower mortality (17.4% vs. 36.1%, *p* = 0.0475) in the St-LBBB group. Lower VA incidence in NICM with St-LBBB (*p* = 0.049) but not in ICM with St-LBBB (*p* = 0.25). Benefit concentrated in the non-ischemic subgroup.	Positive

Abbreviations: CI, confidence interval; ECG, electrocardiogram; LBBB, left bundle branch block; LVAD, left ventricular assist device; LVEF, left ventricular ejection fraction; LVESV, left ventricular end-systolic volume; ICM, ischemic cardiomyopathy; NICM, non-ischemic cardiomyopathy; VA, ventricular arrhythmia; HR, hazard ratio; OR, odds ratio; CMR, cardiac magnetic resonance; HF, heart failure; NYHA, New York Heart Association; St-LBBB, Strauss-defined left bundle branch block; Defining, the original Strauss criteria paper (no outcome comparison).

**Table 2:** Part 2: Randomized trials of conduction system pacing (CSP) versus biventricular pacing (BiVP) in populations enriched for Strauss-type or typical LBBB morphology (2021–2026). Note: LEVEL-AT also enrolled patients with non-LBBB wide QRS ≥ 150 ms; PhysioSync-HF required LBBB with QRS ≥ 130 ms but did not formally mandate strict Strauss criteria; LEFT-BUNDLE-CRT used Strauss criteria and a response-based primary endpoint (not time-to-event), and is shown separately at the bottom of the table.

Trial/Year	Design and Patients	CSP Approach and Technical Success	Primary Result	Verdict
His-Alternative [33]	RCT, single center (Denmark) *n* = 50 LVEF ≤ 35%, Strauss LBBB Follow-up: 6 months	His bundle–corrective CRT vs. BiVP His-corrective capture confirmed in 72% of HBP arm; 28% could not be corrected	ITT analysis: ΔLVEF +16% His-CRT vs. +13% BiVP (*p* = 0.25); non-significant for all endpoints. Per-protocol (confirmed capture only): LVEF 48% vs. 42% BiVP (*p* < 0.05); LVESV 65 vs. 83 mL (*p* < 0.05). First RCT to use Strauss criteria as the sole LBBB enrollment criterion.	Positive (per-protocol)
LBBP-RESYNC [34]	RCT, 2 centers (China) *n* = 40 (NICM only) Strauss LBBB; LVEF ≤ 40% Follow-up: 6 months	LBBP vs. BiVP Crossover to BiVP allowed if LBBP failed LBBP technical success: 90%	LBBP produced significantly greater LVEF increase (mean difference 5.6%; 95% CI 0.3–10.9; *p* = 0.039), larger LVESV reduction (−24.97 mL; 95% CI −49.58 to −0.36), and lower NT-proBNP than BiVP. NYHA class, 6MWD, QRS duration, and CRT response rates were comparable between groups. NICM-only population; most biologically homogeneous CSP trial in this series.	Positive
LEVEL-AT [35]	RCT, single center (Spain) *n* = 70 (Strauss LBBB subgroup + non-LBBB patients) Primary endpoint at 45 days; clinical follow-up 6 months	CSP (HBP or LBBAP) vs. BiVP 23% crossed over from CSP to BiVP when initial placement failed	CSP non-inferior to BiVP for change in LV activation time by ECGi at 45 days (primary endpoint). Similar LV reverse remodeling and death or HF hospitalization at 6 months.	Non-inferior
HeartSync-LBBP [36]	RCT, 6 centers (China) *n* = 200 (82.5% NICM) Complete LBBB; LVEF ≤ 35% Median follow-up: 36 months	LBBP vs. BiVP LBBB correction confirmed at implantation All operators > 285 prior CSP cases LBBP success: 98%; BiVP success: 94%	Death or HF hospitalization: 8% LBBP vs. 28% BiVP (HR 0.26; 95% CI 0.12–0.57). Driven by HF hospitalization reduction (7% vs. 28%; HR 0.23). All-cause mortality: 2% vs. 5% (NS). Super-response rate higher with LBBP (55% vs. 36%; *p* = 0.007). Largest trial with the longest follow-up in a population enriched for physiologically correctable LBBB; procedural success was high.	Positive (superior)
PhysioSync-HF [37]	RCT, 14 centers (Brazil) *n* = 173 (~67% DCM) LBBB, QRS ≥ 130 ms, LVEF ≤ 35% 96.6% (CSP)/94.2% (BiVP) had Strauss-type LBBB at baseline; strict criteria not formally mandated Median follow-up: 12 months	CSP (LBBAP preferred; also deep septal and HBP accepted) vs. BiVP CSP success: 69%; ~20% received deep septal, not true LBB capture 42.8% of implants by operators with <40 prior CSP cases	Primary hierarchical composite (death, HF hospitalization, urgent HF visits, change in LVEF) favored BiVP (OR 2.36; 95% CI 1.37–4.06; *p* = 0.99 for non-inferiority; *p* = 0.002 for between-group difference). Time-to-event composite: HR 2.35 (95% CI 0.99–5.61). Mean LVEF at 12 months: 35% CSP vs. 39% BiVP (mean difference 3.8%; 95% CI 0.3–7.3%). NYHA class, QRS duration, KCCQ, and natriuretic peptides improved comparably in both groups. High heterogeneity in CSP delivery (69% success); 42.8% of implants by operators with <40 prior CSP cases. Lower procedural success and less experienced operators may have influenced outcomes; see accompanying editorial.	Negative (limited by heterogeneous CSP delivery and variable procedural success)
LEFT-BUNDLE-CRT [38]	RCT, multi-center (Spain) *n* = 176 Strauss LBBB; class I/IIa CRT indication Follow-up: 12 months (6-month primary endpoint)	LBBAP-CRT vs. BiVP Crossover in 26 patients (14.9%)	Primary endpoint (CRT response at 6 months: improved CCS or ≥15% LVESV reduction): 94.6% BiVP vs. 89.7% LBBAP (RR 0.95; 95% CI 0.88–1.02)—did NOT meet non-inferiority. CCS improvement: 77% BiVP vs. 68% LBBAP. LVESV reduction ≥ 15%: 85% BiVP vs. 79% LBBAP. HF hospitalization and adverse events: comparable between groups. First European multicenter RCT in a Strauss-defined LBBB population.	Non-inferior not shown (comparable clinical outcomes)

Abbreviations: 6MWD, 6 min walk distance; CCS, clinical composite score; CI, confidence interval; CRT, cardiac resynchronization therapy; DCM, dilated cardiomyopathy; ECGi, electrocardiographic imaging; HBP, His bundle pacing; HF, heart failure; HR, hazard ratio; ITT, intention-to-treat; KCCQ, Kansas City Cardiomyopathy Questionnaire; LBBAP, left bundle branch area pacing; LBBP, left bundle branch pacing; LV, left ventricular; LVEF, left ventricular ejection fraction; LVESV, left ventricular end-systolic volume; NICM, non-ischemic cardiomyopathy; NS, not significant; NT-proBNP, N-terminal pro-B-type natriuretic peptide; NYHA, New York Heart Association functional class; OR, odds ratio; RCT, randomized controlled trial; RR, relative risk; ΔLVEF, change in left ventricular ejection fraction.

## Data Availability

No new data were created or analyzed in this study. Data sharing is not applicable to this article.

## Supplementary Materials

The following supporting information can be downloaded at: https://www.mdpi.com/article/10.3390/jpm16070389/s1, Table S1: Quality assessment of observational studies (Part 1) using the Newcastle-Ottawa Scale; Table S2: Quality assessment of randomized controlled trials (Part 2) using the Cochrane Risk-of-Bias Tool version 2 (RoB 2). Table S3: full electronic search strategies (PubMed, EMBASE, Cochrane CENTRAL); Table S4: completed PRISMA 2020 checklist.
