# Supplementary material for: True Left Bundle Branch Block by Strauss Criteria: Impact on CRT Response and Conduction System Pacing Trial Design—A Systematic Evidence Synthesis Toward Personalized Patient Selection
Source: J Pers Med. 2026 Jul 21;16(7):389. doi: 10.3390/jpm16070389 (PMC13413429; doi:10.3390/jpm16070389)
Supplement: Supplementary file 1 [file jpm-16-00389-s001.zip › jpm-4326883-supplementary.pdf]

Supplementary Materials

Table S1: Quality assessment of observational studies (Part 1) using the Newcastle-Ottawa Scale; Table S2: Quality assessment of randomized controlled trials (Part 2) using the Cochrane Risk-of-Bias Tool version 2 (RoB 2). Table S3: full electronic search strategies (PubMed, EMBASE, Cochrane CENTRAL); Table S4: completed PRISMA 2020 checklist.

Table S1. Quality assessment of observational studies (Part 1) using the Newcastle-Ottawa Scale.

Study	S1	S2	S3	S4	C1	O1	O2	O3	Total /9	Notes
Mascioli 2012	★	★	★	★	★	★	★	★	**8**	
Risum 2013 (Am Heart J)	★	★	★	★	★★	★	★	★	**9**	
Tian 2013	★	★	★	★	★	★	★	☆	**7**	*Short follow-up*
Jackson 2014	★	★	★	★	★★	★	★	★	**9**	
Emerek 2015	★	★	★	★	★	★	★	☆	**7**	
Risum 2015 (JACC)	★	★	★	★	★★	★	★	★	**9**	
Bertaglia 2017 (CRT MORE)	★	★	★	★	★	★	★	★	**8**	*Multicenter registry*
Garcia-Seara 2018	★	★	★	★	★	★	★	★	**8**	
Kashtanova 2018	★	☆	★	★	★	★	★	☆	**6**	*Small sample (n=39)*
Caputo 2018	★	★	★	★	★	★	★	★	**8**	
Jastrzebski 2018	★	★	★	★	★★	★	★	★	**9**	*Longest follow-up (9 yrs)*
Hadjis 2019	★	★	★	★	★	★	★	☆	**7**	
van Stipdonk 2020 (JACC CE)	★	★	★	★	★★	★	★	★	**9**	*Largest dataset (n=1,492)*
Bouazzi 2021	★	★	★	★	★	★	★	★	**8**	
Shoman 2022	★	★	★	★	★	★	★	☆	**7**	*Small Strauss subgroup*
Mugnai 2022	★	★	★	★	★	★	★	★	**8**	

Saplaouras 2025	★	★	★	★	★	★	★	★	8	*Authors' own cohort*

★ = criterion met; ☆ = criterion not met. S1: representativeness of the exposed cohort. S2: selection of the non-exposed cohort from the same community. S3: ascertainment of exposure (Strauss LBBB classification). S4: demonstration that outcome was absent at study start. C1: comparability of cohorts on the basis of study design or analysis (★★ = adjustment for ≥2 confounders including ischemic vs non-ischemic etiology; ★ = adjustment for at least one confounder). O1: assessment of outcome (blinded or record-linkage). O2: follow-up sufficient for outcomes to occur (minimum 6 months). O3: adequacy of follow-up (completeness ≥80%). Total score: 7–9 = high quality; 5–6 = moderate quality.

Table S2. Quality assessment of randomized controlled trials (Part 2) using the Cochrane Risk-of-Bias Tool version 2 (RoB 2).

**Trial**	**D1 Randomiz-ation**	**D2 Intervention deviations**	**D3 Missing outcome data**	**D4 Outcome measurement**	**D5 Selective reporting**	**Overall**	**Key rationale**
His-Alternative Vinther 2021	Low	Some concerns	Low	Low	Low	**Some concerns**	*Operator blinding not feasible in device implantation; reliance on per-protocol findings limits interpretation beyond the ITT analysis (D2).*
LBBP-RESYNC Wang 2022	Low	Some concerns	Low	Low	Low	**Some concerns**	*Single-blind design with operators aware of assignment; small sample size (n=40) increases the risk of chance baseline imbalance.*
LEVEL-AT Pujol-Lopez 2022	Low	Some concerns	Low	Low	Low	**Some concerns**	*23% crossover from CSP to BiVP; operators were aware of treatment assignment.*
HeartSync-LBBP Chen 2026	Low	Low	Low	Low	Low	**Low**	*LBBB correction confirmed at implantation; all operators >285 prior CSP cases; outcomes independently adjudicated by blinded committee. Population enriched for true, physiologically correctable LBBB.*

PhysioSync-HF Zimerman 2026	Low	Some concerns	Low	Low	Low	**Some concerns**	*Approximately 20% of patients received deep septal pacing without confirmed LBB capture; 42.8% of implants performed by operators with fewer than 40 prior CSP cases; blinded outcome committee mitigates D4. Inclusion justified post-hoc: 96.6%/94.2% of patients had Strauss-type LBBB at baseline.*
LEFT-BUNDLE-CRT Cano 2026	Low	Some concerns	Low	Low	Low	**Some concerns**	*Operator blinding not feasible in device implantation; 14.9% crossover rate from LBBAP to BiVP; non-inferiority response-based primary endpoint. Strauss-defined LBBB used as formal enrollment criterion (D2).*

RoB 2 domains: D1 = bias from the randomization process; D2 = bias due to deviations from intended interventions (blinding of participants and personnel); D3 = bias due to missing outcome data; D4 = bias in measurement of the outcome; D5 = bias in selection of the reported result. Operator blinding is structurally not feasible in device implantation trials. D2 ratings of "Some concerns" reflect the inherent inability to blind operators in device implantation trials and the presence of protocol-defined crossover or heterogeneous CSP delivery, rather than a correctable methodological flaw or uncontrolled deviation from assigned treatment.

Table S3. Full electronic search strategies (PubMed, EMBASE, and the Cochrane Central Register of Controlled Trials).The initial search was run in December 2025 and updated in April 2026. No language or date restrictions were applied; reference lists of all included articles were hand-searched. Two concept blocks were combined with AND: (A) left bundle branch block / Strauss criteria, and (B) cardiac resynchronization therapy / conduction system pacing.

1. PubMed (MEDLINE) — https://pubmed.ncbi.nlm.nih.gov

```
#1  "Bundle-Branch Block"[Mesh]
#2  ("left bundle branch block"[tiab] OR LBBB[tiab])
#3  (Strauss[tiab] OR "strict LBBB"[tiab] OR "true LBBB"[tiab] OR "complete LBBB"[tiab]
     OR "strict left bundle branch block"[tiab] OR "true left bundle branch block"[tiab]
     OR "complete left bundle branch block"[tiab])
#4  #1 OR #2 OR #3
#5  "Cardiac Resynchronization Therapy"[Mesh]
#6  ("cardiac resynchronization therapy"[tiab] OR "cardiac resynchronisation therapy"[tiab]
     OR CRT[tiab] OR "biventricular pacing"[tiab] OR BiVP[tiab])
#7  ("conduction system pacing"[tiab] OR CSP[tiab] OR "left bundle branch pacing"[tiab]
     OR LBBP[tiab] OR "left bundle branch area pacing"[tiab] OR LBBAP[tiab]
     OR "His bundle pacing"[tiab] OR "His-bundle pacing"[tiab])
#8  #5 OR #6 OR #7
#9  #4 AND #8
```

2. EMBASE (Ovid)

```
1   exp bundle branch block/
2   (left bundle branch block or LBBB).ti,ab,kw.
3   (Strauss or "strict LBBB" or "true LBBB" or "complete LBBB" or strict left bundle
     branch block or true left bundle branch block or complete left bundle branch
block).ti,ab,kw.
4   1 or 2 or 3
5   exp cardiac resynchronization therapy/
6   (cardiac resynchron* therapy or CRT or biventricular pacing or BiVP).ti,ab,kw.
7   (conduction system pacing or CSP or left bundle branch pacing or LBBP or left bundle
     branch area pacing or LBBAP or His bundle pacing).ti,ab,kw.
8   5 or 6 or 7
9   4 and 8
```

3. Cochrane CENTRAL (Wiley) — https://www.cochranelibrary.com

```
#1  MeSH descriptor: [Bundle-Branch Block] explode all trees
#2  (left bundle branch block or LBBB):ti,ab,kw
#3  (Strauss or "strict LBBB" or "true LBBB" or "complete LBBB"):ti,ab,kw
#4  #1 OR #2 OR #3
#5  MeSH descriptor: [Cardiac Resynchronization Therapy] explode all trees
#6  ("cardiac resynchronization therapy" or "cardiac resynchronisation therapy"
     or CRT or "biventricular pacing"):ti,ab,kw
#7  ("conduction system pacing" or CSP or "left bundle branch pacing" or LBBP
     or "left bundle branch area pacing" or LBBAP or "His bundle pacing"):ti,ab,kw
#8  #5 OR #6 OR #7
#9  #4 AND #8
```

Field tags: [tiab] = title/abstract (PubMed); .ti,ab,kw. = title, abstract, keyword (Ovid EMBASE); :ti,ab,kw = title, abstract, keyword (Cochrane). Truncation (*) captures spelling variants. After deduplication the combined search yielded 312 records, consistent with the PRISMA 2020 flow diagram (Figure 1).

Table S4. Completed PRISMA 2020 checklist (Page MJ, et al. BMJ 2021;372:n71). *Items not applicable to this systematic evidence synthesis (no meta-analysis, no protocol registration, no formal certainty grading) are marked accordingly, consistent with the Methods.*

#	Checklist item	Location where item is reported / note
**TITLE**		
**1**	Title — identify the report as a systematic review.	Title page ("... A Systematic Evidence Synthesis ...").
**ABSTRACT**		
**2**	Abstract — structured summary (per PRISMA 2020 for Abstracts).	Abstract.
**INTRODUCTION**		
**3**	Rationale for the review.	Introduction (paragraphs 1–3).
**4**	Explicit objective(s)/question(s).	Introduction (final paragraph).
**METHODS**		
**5**	Eligibility criteria and grouping.	Methods 2.1 (Part 1 and Part 2 inclusion criteria).
**6**	Information sources and last-search dates.	Methods (PubMed, EMBASE, Cochrane; Dec 2025, updated Apr 2026).
**7**	Full search strategies.	Methods; Table S3.
**8**	Selection process.	Methods (two reviewers; disagreements resolved by a third).
**9**	Data collection process.	Methods (narrative extraction into Tables 1–2).
**10a**	Outcomes sought.	Methods; Tables 1–2.
**10b**	Other variables sought.	Tables 1–2.
**11**	Risk-of-bias assessment methods.	Methods; Tables S1 and S2.
**12**	Effect measures.	Results; Tables 1–2.
**13a**	Study grouping for synthesis.	Methods (Part 1 observational; Part 2 RCTs).
**13b**	Data preparation.	Not applicable — narrative synthesis.
**13c**	Tabulation/display.	Tables 1–2; Figure 1.
**13d**	Synthesis/meta-analysis method.	Methods — narrative synthesis; no meta-analysis (heterogeneity).
**13e**	Exploration of heterogeneity.	Discussion (by aetiology and operator experience).
**13f**	Sensitivity analyses.	Not applicable.
**14**	Reporting-bias assessment.	Limitations (qualitative; no funnel plot).
**15**	Certainty (GRADE) assessment.	Not performed; stated in Methods.
**RESULTS**		
**16a**	Selection results (flow).	Results; Figure 1 (312 → 42 → 24).
**16b**	Excluded studies with reasons.	Methods/Results.
**17**	Study characteristics.	Tables 1 and 2.
**18**	Risk of bias per study.	Tables S1 and S2; Methods summary.
**19**	Results of individual studies.	Tables 1 and 2.
**20a**	Syntheses: contributing studies.	Results; Methods summary.
**20b**	Statistical syntheses.	Not applicable — no meta-analysis.
**20c**	Causes of heterogeneity.	Discussion.
**20d**	Sensitivity analyses.	Not applicable.
**21**	Reporting biases per synthesis.	Limitations.
**22**	Certainty per outcome.	Not assessed (no GRADE).
**DISCUSSION**		
**23a**	Interpretation in context.	Discussion.
**23b**	Limitations of the evidence.	Limitations.
**23c**	Limitations of review processes.	Limitations.
**23d**	Implications for practice/research.	Discussion/Conclusions.
**OTHER INFORMATION**		

**24a**	Registration information.	Methods — not prospectively registered.
**24b**	Where protocol can be accessed.	Methods — no protocol prepared.
**24c**	Amendments.	Not applicable.
**25**	Support/funding.	Funding statement — no external funding.
**26**	Competing interests.	Conflicts of Interest — none declared.
**27**	Availability of data/materials.	Data Availability Statement; Tables 1–2 and S1–S2.
